# Investigation of the mechanism of *Prunella vulgaris* in treatment of papillary thyroid carcinoma based on network pharmacology integrated molecular docking and experimental verification

**DOI:** 10.1097/MD.0000000000033360

**Published:** 2023-04-28

**Authors:** Xiling Zhu, Yan Li, Xiaodong Wang, Yuanshe Huang, Jingxin Mao

**Affiliations:** a Anshun University, Guizhou Anshun, China; b Chongqing Medical and Pharmaceutical College, Chongqing, China; c College of Pharmaceutical Sciences, Southwest University, Chongqing, China.

**Keywords:** molecular docking, network pharmacology, papillary thyroid carcinoma, *Prunella vulgaris*, PTC

## Abstract

To analyze the molecular mechanism of *Prunella vulgaris* L. (*PV*) in the treatment of papillary thyroid carcinoma (PTC) by using network pharmacology combined with molecular docking verification. Traditional Chinese Medicine Systems Pharmacology Database and Analysis Platform database was used to predict the main active components of *PV*, Traditional Chinese Medicine Systems Pharmacology Database and Analysis Platform, PubChem, and Swiss Target Prediction databases were used to obtain the corresponding targets of all active components. Targets collected for PTC treatment through Gene Cards, Digest and Online Mendelian Inheritance in Man databases respectively. The Search Tool for the Retrieval of Interaction Gene/Protein database was used to obtain the interaction information between proteins, and the topology analysis and visualization were carried out through Cytoscape 3.7.2 software (https://cytoscape.org/). The R package cluster profiler was used for gene ontology and Kyoto encyclopedia of genes and genomes analysis. The “active ingredient-target-disease” network was constructed by using Cyto scape 3.7.2, and topological analysis was carried out to obtain the core compound. The molecular docking was processed by using Discovery Studio 2019 software, and the core target and active ingredient were verified. The inhibition rate was detected by CCK8 method. Western blot was used to detect the expression levels of kaempferol anti-PTC related pathway proteins. A total of 11 components and 83 corresponding targets in the component target network of *PV*, of which 6 were the core targets of *PV* in the treatment of PTC. It was showed that quercetin, luteolin, beta (β)-sitosterol, kaempferol may be the core components of *PV* in the treatment of PTC. vascular endothelial growth factor A, tumor protein p53, transcription factor AP-1, prostaglandin endoperoxidase 2, interleukin 6, and IL-1B may be important targets for the treatment of PTC. The main biological processes mainly including response to nutrient levels, response to xenobiotic stimulus, response to extracellular stimulus, external side of plasma membrane, membrane raft, membrane microdomain, serine hydrolase activity, serine-type endopeptidase activity, antioxidant activity, etc IL-17 signaling pathway, and PI3K-Akt signaling pathway may affect the recurrence and metastasis of PTC. Kaempferol may significantly reduce the activity of Papillary cells of human thyroid carcinoma bcpap cell lines cells compared with quercetin, luteolin, β-sitosterol. Kaempferol may reduce the protein expression levels of interleukin 6, vascular endothelial growth factor A, transcription factor AP-1, tumor protein p53, 1L-1B and prostaglandin endoperoxidase 2, respectively. *PV* has the characteristics of multi-components, multi-targets and multi- pathways in the treatment of PTC, which network pharmacology help to provides a theoretical basis for the screening of effective components of *PV* and further research.

## 1. Introduction

Globally, among the malignant tumors of the thyroid endocrine system, thyroid cancer has a high incidence rate, accounting for more than 80% to 90% of all endocrine tumors.^[1]^ Among thyroid cancers, papillary thyroid cancer (PTC) has the highest incidence rate, accounting for more than 80% of thyroid malignant tumors. The formation of PTC is a very complex biochemical process, characterized by the abnormality of various molecules.^[2]^ Large scale genome research shows that genetic changes play a key role in the occurrence and development of PTC, but the reasons for its continued high incidence are not yet clear.^[3]^ Thyroid disease screening has become a routine examination item of health examination, but there is still a lack of thyroid disease screening in some people’s health examination items, and some people do not have the awareness of regular health examination, and they do not go to the hospital until they find a neck lump or a symptom of neck discomfort.^[4]^ At present, total thyroidectomy, radioiodine therapy and thyrotropin suppression therapy have been highly developed and widely accepted.^[5]^

The adverse reaction of Traditional Chinese Medicine (TCM) is relatively small, which can help reducing the recurrence and metastasis of tumors, improving the quality of life of patients, with obvious advantages.^[6]^ *Prunella vulgaris* L. (*PV*), a traditional Chinese herbal medicine in China, is a plant of *Labiatae*. It is distributed around the world, mainly in temperate and tropical mountains, such as Europe, Asia, northwest Africa and North America. It is mainly distributed in the Huaihe River basin and the middle and lower reaches of the Yangtze River basin in China.^[7]^ According to the Pharmacopoeia of the People’s Republic of China, it has the effects of clearing the liver, purging fire, improving eyesight, dispersing knots, and reducing swelling in clinical efficacy.^[8]^ Therefore, *PV* is also commonly used in clinical treatment of thyroid cancer, breast cancer, liver injury, hypertension, hyperglycemia, prostate diseases, and other diseases.^[9–12]^

*PV* mainly contains terpenoids, phenolic acids, organic acids, sterols, coumarins, flavonoids, polysaccharides, and volatile oils.^[10]^ Modern pharmacological studies have shown that its extracts have a variety of biological activities, such as antioxidation, regulating immune function, anti-inflammatory, antibacterial, antiviral, and regulating the microenvironment of tumor metastasis.^[10,11]^ With the extensive clinical application of *PV*, the experimental research of *PV* has gradually deepened, and its pharmacological effects and molecular mechanism of action are gradually clear. However, the active ingredients contained in *PV* are relatively complex, and its main medicinal substances and pharmacological mechanism are still unclear. The inhibition of thyroid cancer, active ingredients and potential action targets of *PV* are still lack of systematic research.

Network pharmacology can successfully establish the relationship among “drugs-components-targets-diseases” and reveal the complex functions of Chinese medicine at multiple levels and systems.^[13]^ The network pharmacology technology can be used to obtain the relationship between “multi components and multi targets” and protein interaction, predict the TCM and its molecular mechanism of action, and make the research of TCM extend better. This study adopts network pharmacology combined molecular docking methods to explore the effective components and potential mechanism of action of *PV* in inhibiting PTC, so as to provide more theoretical support for clinical use of *PV* in treating PTC.

## 2. Materials and Methods

### 2.1. Prediction of main active components and corresponding targets of *PV*

Using Traditional Chinese Medicine Systems Pharmacology Database and Analysis Platform (TCMSP) (http://tcmspw.com/tcmsp.php) to search the chemical components of *PV*, select the components with oral bioavailability≥30% and drug like property≥0.18 as the effective components, download the corresponding chemical structure, and save it in MOL2 format.

### 2.2. Construction of the relationship of active ingredient and disease target

Through TCMSP database, Swiss Target Prediction (http://www.swisstargetprediction.ch/) database and PubChem database (https://pubchem.ncbi.nlm.nih.gov/) respectively. The target library corresponding to the main active components of *PV* was obtained by searching the corresponding targets of all active components in *PV* and removing duplicate genes. Using the Gene Cards database (https://www.genecards.org/), Disgenet database (https://www.disgenet.org/), and Online Mendelian Inheritance in Man database (https://omim.org/) respectively to collects targets for PTC treatment with the key word “papillory thyroid cancer” OR “PTC,” removes duplicate genes, and obtains the corresponding target library.

### 2.3. Protein-protein interaction (PPI) network construction

In order to further study the action mechanism of *PV* action target and disease target at the protein level, we use R language to obtain the intersection between them, and use R package Venn Diagram for visualization. Input the intersection gene into Search Tool for the Retrieval of Interaction Gene/Protein database (https://string-db.org/) to obtain the interaction information between proteins, and conduct topological analysis and visualization through R language.

### 2.4. Gene ontology (GO) and Kyoto encyclopedia of genes and genomes (KEGG) analysis

In order to further study the core target function and the main action pathway of *PV* in the treatment of thyroid cancer, the R package cluster profiler was used to conduct GO and KEGG analysis on the intersection targets. The threshold values were set at *P*<.05, *q*<0.05, and finally 20 results with the lowest *P* value were retained for visualization. To validated the anti-PTC mechanism of *PV* across the key targets and multiple pathways, the KEGG mapper functional analysis was utilized to mark the target genes on the pathway associated with PTC.

### 2.5. Build a network of “active ingredient -target-disease”

In order to analyze the correlation between active compounds, a network of “active ingredient-target-disease” was constructed by using Cytoscape 3.7.2 software (https://cytoscape.org/). The function of “network analyzer” is used for topological analysis to obtain the core active ingredients.

### 2.6. Molecular docking

In order to ensure the reliability of molecular docking, the protein crystal structure with resolution < 2.5 Å and ligand complex is selected to establish a molecular docking model. The molecular docking program uses the Libdock module of the Discovery Studio 2019 software package. As a small molecule ligand, the key active ingredient CAS number is searched through the PubChem database, derived from Chem 3-dimensional 19.0, and the lowest energy is calculated and saved in MOL2 format. As the receptor, the core target protein is downloaded from the protein data bank (PDB) database in PDB format. First, the water molecules in the PDB structure are removed, and the docking active pocket is defined by the original ligand molecules. After setting the docking parameters, the ligand molecules in the crystal structure are extracted and re docked to the predefined active pocket. At the same time, the root mean square difference between the docked ligand molecule conformation and the initial conformation in the crystal structure is calculated. If the root means square difference value is <2.5, the molecular docking result is considered reliable. On this basis, molecular docking was performed on the core target and corresponding compounds to verify the binding strength of the core target and the main active components of *PV*.

### 2.7. Experimental verification

#### 2.7.1. Materials and instruments.

Human thyroid papillary carcinoma cell Papillary cells of human thyroid carcinoma bcpap cell lines (BCPAP) was purchased from the Shanghai Cell Bank of the Chinese Academy of Sciences. Quercetin (CAS No. 117-39-5), luteolin (CAS No. 491-70-3), beta-sitosterol (β-sitosterol) (CAS No. 83-46-5), kaempferol (CAS No. 520-18-3) were purchased from Selleck China company respectively. One thousand six hundred-forty culture medium, dimethyl sulfoxide (DMSO), fetal bovine serum, penicillin streptomycin double antibody, 0.25% trypsin, cell counting kit 8 (CCK8), and DMSO purchased from Sangon Biotech (Shanghai) Co., Ltd. RIPA Lysis Buffer, rabbit derived vascular endothelial growth factor A (VEGFA) antibody, tumor protein p53 (TP53) antibody, transcription factor AP-1 (JUN) antibody, prostaglandin endoperoxidase 2 (PTGS2) antibody, interleukin 6 (IL6) antibody, IL-1B antibody, rabbit derived House keeping protein. (glyceraldehyde-3-phosphate dehydrogenase) antibody and goat anti-rabbit IgG were purchased from Beyotime Biotechnology Co., Ltd. and ThermoFisher Scientific Co., Ltd. respectively. The instrument includes BioRad full-automatic microplate reader, BioRad chemiluminescence gel imaging system, OLYMPUS IX51 inverted microscope, MCO-15AC SANYO CO_2_ constant temperature incubator, Eppendorf 5702R low-speed centrifuge.

#### 2.7.2. CCK8 assay kit.

BCPAP cell was cultured in 1640 medium containing 10% fetal bovine serum and 1% penicillin streptomycin at 37°C and 5% CO_2_ incubator. Cell growth was observed under inverted microscope, and cells in logarithmic growth phase were taken for subsequent experiments. Grouping method: different concentration of drug group including: 2.5, 5, 10, 20, 40, 80, 160 µmol/L, 4 active compounds quercetin, luteolin, β-sitosterol, kaempferol of *PV* respectively. Control group: the same volume of DMSO (0.01%). BCPAP cells were treated with 5 × 10^3^ cells (100 μL suspension) was inoculated into 96 well plates. Place it in a 37°C, 5% CO_2_ incubator for 24 hours, and then the cell monolayer will cover the bottom of the hole. The concentration of 4 active compounds added to the cells at 2.5, 5, 10, 20, 40, 80, 160 µmol/L in complete medium 100 μL, each with 3 double wells respectively. Add 90 μL serum free 1640 medium and 10 μL CCK8 solution per hole, continue to culture for 2 hours, and then measure the absorbance of each hole with the microplate reader at 450 nm wavelength. Repeat the experiment for 3 times. Cells were continued to culture for 48 and 72 hours, and measured the cell activity in each group at 24, 48, 72 hours respectively. Measured the OD value of each well on the computer for the sample to be tested. The cell survival rate is expressed as a percentage, and calculate the half inhibition rate (IC_50_).

#### 2.7.3. Western blot assay kit.

Western blot method was used to detect the expression levels of anti-PTC related proteins. Kaempferol with the final concentration of 10, 20, 40, 80 µmol/L was added to BCPAP cells, culture for 48 hours, collect cells on ice, extract the total protein of each group of cells by RIPA lysis buffer assay kit. Measure the protein concentration by BCA method, gradually carry out sample loading, electrophoresis, membrane transfer, membrane washing, and sealing. Add the corresponding primary antibody for incubation at room temperature for 1 hour according to instructions, overnight at 4 °C, add the secondary antibody after membrane washing, incubate in a shaking table for 2 hours, and wash the membrane with TBST for 3 times, 10 minutes each time. ECL imaging system is used for luminous development. The experiment is repeated at least 3 times, and the gray value is measured and statistically analyzed.

### 2.8. Statistical methods

The data was analyzed by SPSS 20.0 statistical software (SPSS Inc., Chicago, IL), and all the images were drawn by GraphPad Prism 9.0 software (https://www.graphpad.com/). Image J software (https://imagej.net/software/imagej/) was used for protein gray scale calculation, and *P*<.05 was considered as statistically significant difference.

## 3. Results

### 3.1. Main active ingredients of *PV*

According to the screening conditions (oral bioavailability≥30%, drug like property≥0.18), 11 main active ingredients of *PV* were screened (Table 1).

**Table 1:** General information of active ingredients of *PV*.

Mol ID	Molecule Name	MW	OB (%)	DL
MOL000358	Beta-sitosterol	414.79	36.91	0.75
MOL000422	Kaempferol	286.25	41.88	0.24
MOL004355	Spinasterol	412.77	42.98	0.76
MOL000449	Stigmasterol	412.77	43.83	0.76
MOL004798	Delphinidin	303.26	40.63	0.28
MOL000006	Luteolin	286.25	36.16	0.25
MOL006767	Vulgaxanthin-I	339.34	56.14	0.26
MOL006772	Poriferasterol monoglucoside_qt	412.77	43.83	0.76
MOL006774	Stigmast-7-enol	414.79	37.42	0.75
MOL000737	Morin	302.25	46.23	0.27
MOL000098	Quercetin	302.25	46.43	0.28

DL = drug like property, OB = oral bioavailability.

### 3.2. Construction of active ingredient and disease target library

**Figure 1.**
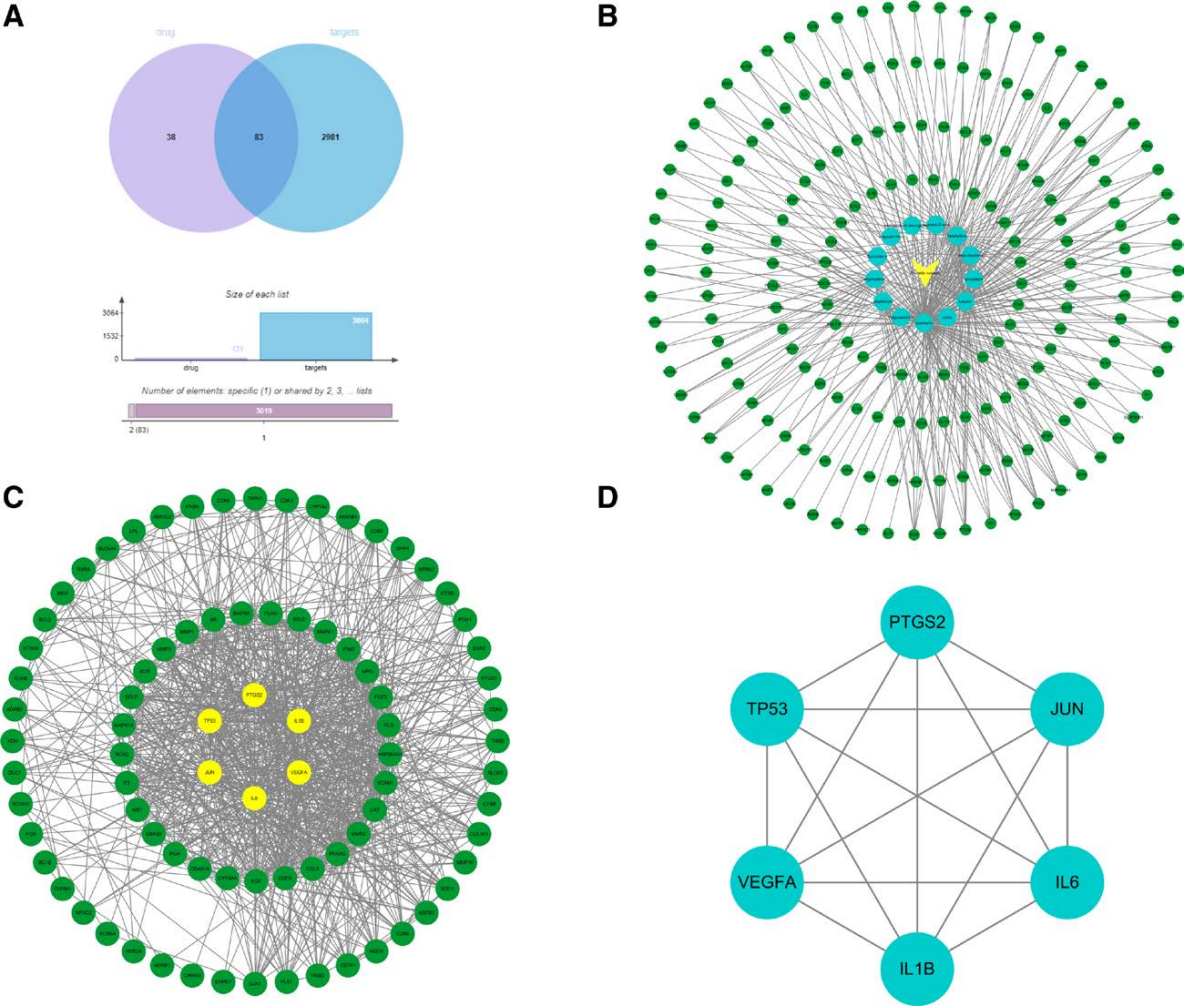
(A). Venn diagram of the common target gene screening of *PV* and PTC related targets. (B) Active ingredients of *PV* anti-PTC target network. (C) PPI network of *PV* on anti-PTC. (D). Key PPI network of *PV* on anti-PTC. PPI = protein-protein interaction, PTC = papillary thyroid cancer.

121 active ingredient targets of *PV* were retrieved through TCMSP, Swiss Target Prediction and PubChem database, and these targets were converted into gene names through UniProt database. Through searching Gene Cards and Online Mendelian Inheritance in Man databases, 3064 PTC related targets were obtained. The R package Venn Diagram was used to finally visualize 83 intersections of active ingredient targets and disease targets (Fig. 1A).

### 3.3. Network diagram of “active components- drugs-target-diseases”

According to the above results, the network of “active components-drugs-target-diseases” was constructed by using Cytoscape 3.7.2 (Fig. 1B). The triangle represents the *PV*, the circle represents the target, and the hexagon represents the effective component of the *PV* monomer compound. Nerwork analyzer analysis shows that quercetin, luteolin, beta (β)-sitosterol and kaempferol have a degree value of the first 4 drugs, which is significantly higher than other effective ingredients (Table 2). At the same time, the network diagram shows that multiple targets are related to different active ingredients, which reflects the characteristics of TCM in treating “multiple-components and multiple-targets.”

**Table 2:** Corresponding core targets genes of 4 main ingredients based on the degree value.

Gene name	Degree	Betweenness centrality	Closeness centrality
IL6	86	0.11175906	0.63176895
IL1B	76	0.09757612	0.60344828
VEGFA	70	0.05093521	0.5794702
TP53	65	0.08084006	0.57003257
EGFR	61	0.08026206	0.57189542
PTGS2	61	0.03475055	0.56451613
EGF	59	0.03509426	0.55031447
JUN	59	0.03786123	0.56634304
CCL2	56	0.01756981	0.54517134
PPARG	50	0.035171	0.53846154
CAT	47	0.0398395	0.53030303
MMP2	47	0.01412851	0.52238806
FGF2	45	0.01865141	0.51928783
APP	41	0.04182184	0.53030303
VCAM1	41	0.00574478	0.50578035
IL2	41	0.01322097	0.50578035
HSP90AA1	40	0.0130669	0.51622419
PLG	39	0.01087161	0.5
IFNG	38	0.010355	0.49715909
MAPK1	36	0.0090428	0.50432277
MPO	35	0.00554788	0.48611111
KDR	33	0.00866159	0.48882682
MAPK8	33	0.0057451	0.49157303
AR	31	0.03269785	0.48476454
MMP3	31	0.00314421	0.4691689
MAPK14	31	0.00361598	0.48882682
NR3C1	30	0.01897313	0.50578035
MMP1	30	0.00273025	0.46542553
SELP	30	0.00859168	0.46296296
SELE	30	0.001925	0.47297297
PLAU	29	0.00640463	0.47169811
CYP3A4	28	0.01779184	0.48882682
F3	27	0.02007971	0.45572917
PGR	26	0.00564901	0.47683924
MET	26	0.01120242	0.47043011
CCL4	26	0.00707492	0.46419098
NOS2	26	0.00761442	0.46542553
GSK3B	25	0.01847344	0.48611111
CD40LG	25	0.00328364	0.45336788
GSTP1	23	0.01372995	0.45454545

IL1B = recombinant human interleukin 1 beta, IL6 = interleukin 6, JUN = transcription factor AP-1, PTGS2 = prostaglandin endoperoxidase 2, TP53 = tumor protein p53, VEGFA = vascular endothelial growth factor A.

### 3.4. PPI network construction

Input 83 intersection targets into Search Tool for the Retrieval of Interaction Gene/Protein database to get the interaction information among targets, and use the software Cytoscape 3.7.2 for topological analysis and visualization to get the interaction network diagram of genes (Fig. 1C). The ordinate represents the target gene name, and the abscissa represents the connectivity. The core network was screened according to the first 6 principles of degree, and the interaction network diagram of 6 core genes was obtained (Fig. 1D).

### 3.5. Result of GO and KEGG analysis

**Figure 2.**
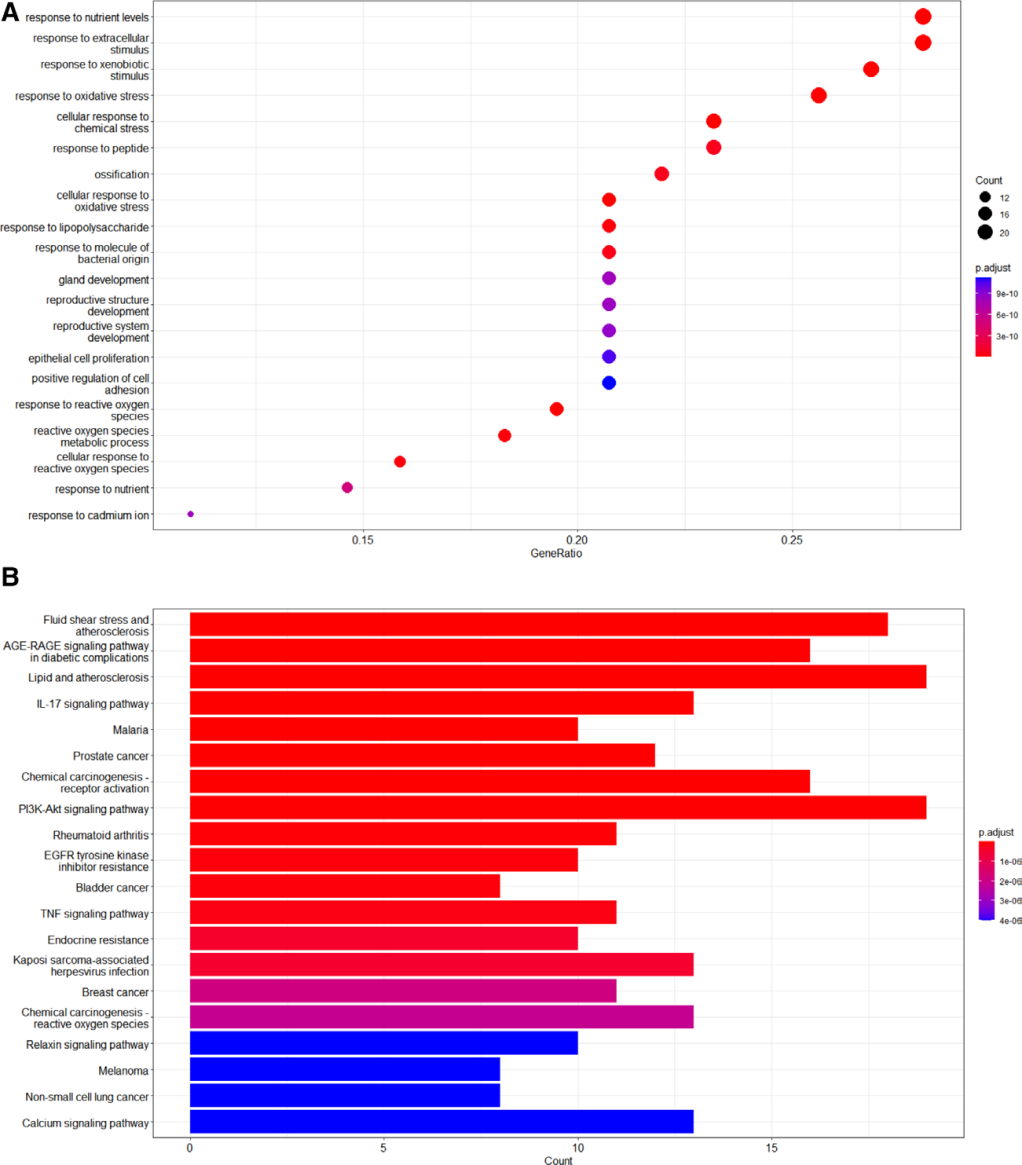
(A). *PV* prevention and treatment of PTC gene GO enrichment analysis bubble diagram. (B) KEGG pathway enrichment histogram chart of the active ingredients of *PV* treatment of PTC. GO = gene ontology, KEGG = Kyoto encyclopedia of genes and genomes, PTC = papillary thyroid cancer.

GO (Fig. 2A) and KEGG (Fig. 2B) analysis of intersection targets are conducted through R package cluster profiler. A total of 1665 GO entries (*q*<0.05) were obtained from GO function enrichment analysis, including 1503 biological process entries, mainly including response to nutrient levels, response to xenobiotic stimulus, response to extracellular stimulus, response to oxidative stress, cellular response to chemical stress, response to reactive oxygen species, cellular response to oxidative stress, response to lipopolysaccharide, reactive oxygen species metabolic process, cellular response to reactive oxygen species etc. A total of 49 cellular composition entries mainly including external side of plasma membrane, membrane raft, membrane microdomain, vesicle lumen, ficolin-1-rich granule lumen, ficolin-1-rich granule, secretory granule lumen, cytoplasmic vesicle lumen, plasma membrane raft, caveola etc. One hundred-thirty molecular function entries mainly including serine hydrolase activity, serine-type endopeptidase activity, antioxidant activity, nuclear receptor activity, ligand-activated transcription factor activity, serine-type peptidase activity, RNA polymerase II CTD heptapeptide repeat kinase activity, protein serine/threonine/tyrosine kinase activity, virus receptor activity, exogenous protein binding etc.

**Figure 3.**
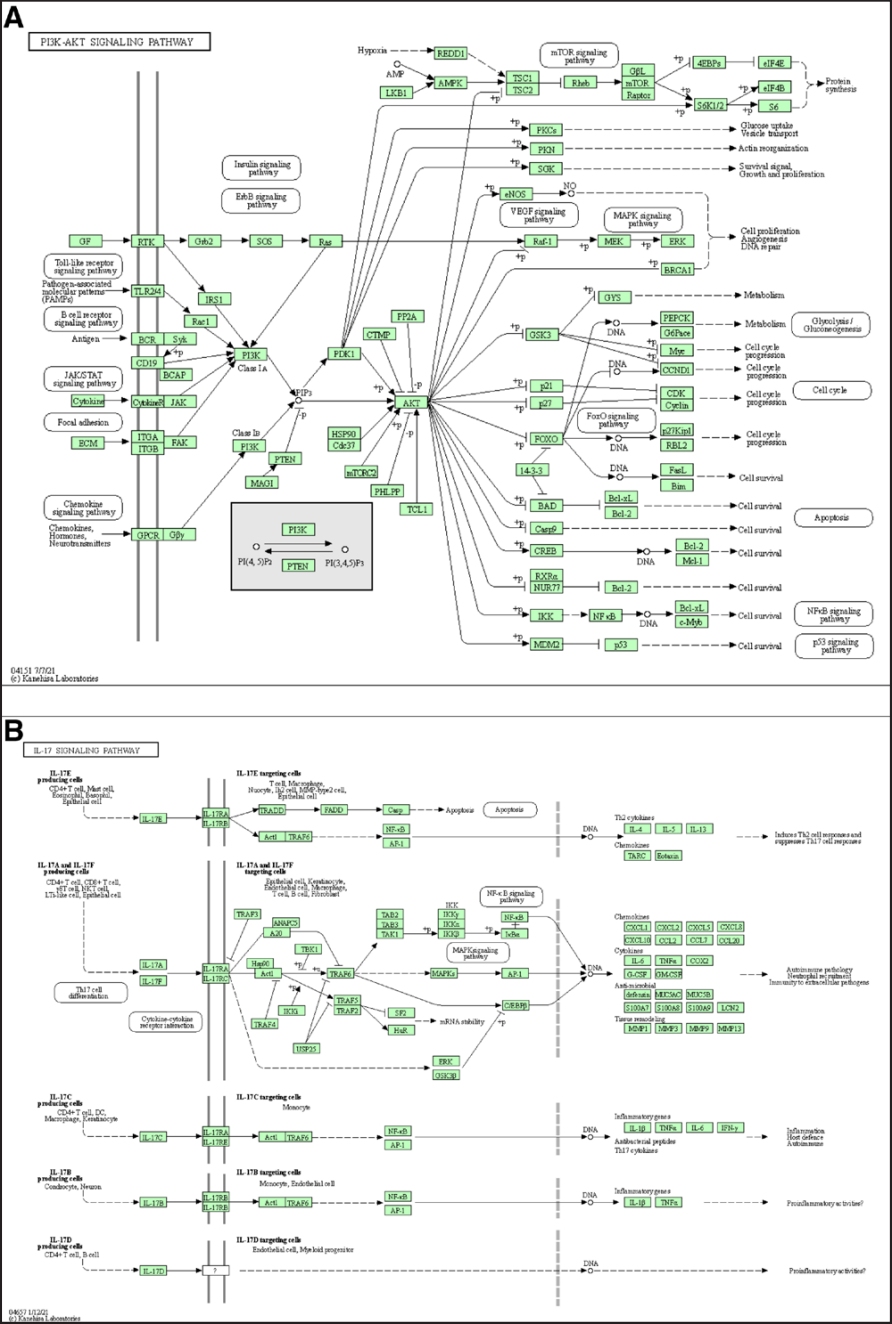
Annotated map of the target genes related the main active components of *PV* on PTC related signaling pathways. (A) IL-17 signaling pathway. (B) PI3K-Akt signaling pathway, PTC = papillary thyroid cancer.

By KEGG pathway analysis (*q*<0.05), 149 pathways were obtained. The first 20 pathways are core pathways, mainly including TFluid shear stress and atherosclerosis, AGE-RAGE signaling pathway in diabetic complications, Lipid and atherosclerosis, IL-17 signaling pathway, Malaria, Prostate cancer, Chemical carcinogenesis-receptor activation, PI3K-Akt signaling pathway, Rheumatoid arthritis, EGFR tyrosine kinase inhibitor resistance, Bladder cancer, TNF signaling pathway, Endocrine resistance, Kaposi sarcoma-associated herpesvirus infection, Breast cancer, Chemical carcinogenesis - reactive oxygen species, Relaxin signaling pathway, Melanoma, Non-small cell lung cancer, Calcium signaling pathway etc. which are mostly related to tumors, indicating that *PV* may treat PTC by acting on different signal pathways. Annotated map of the key target genes locations of *PV* in PTC related pathways was presented in Figure 3. It was revealed that most of the key target genes are associate with IL-17 signaling pathway (Fig. 3A) and PI3K-Akt signaling pathway (Fig. 3B) which may affect the recurrence and metastasis of PTC.

### 3.6. Molecular docking results

**Figure 4.**
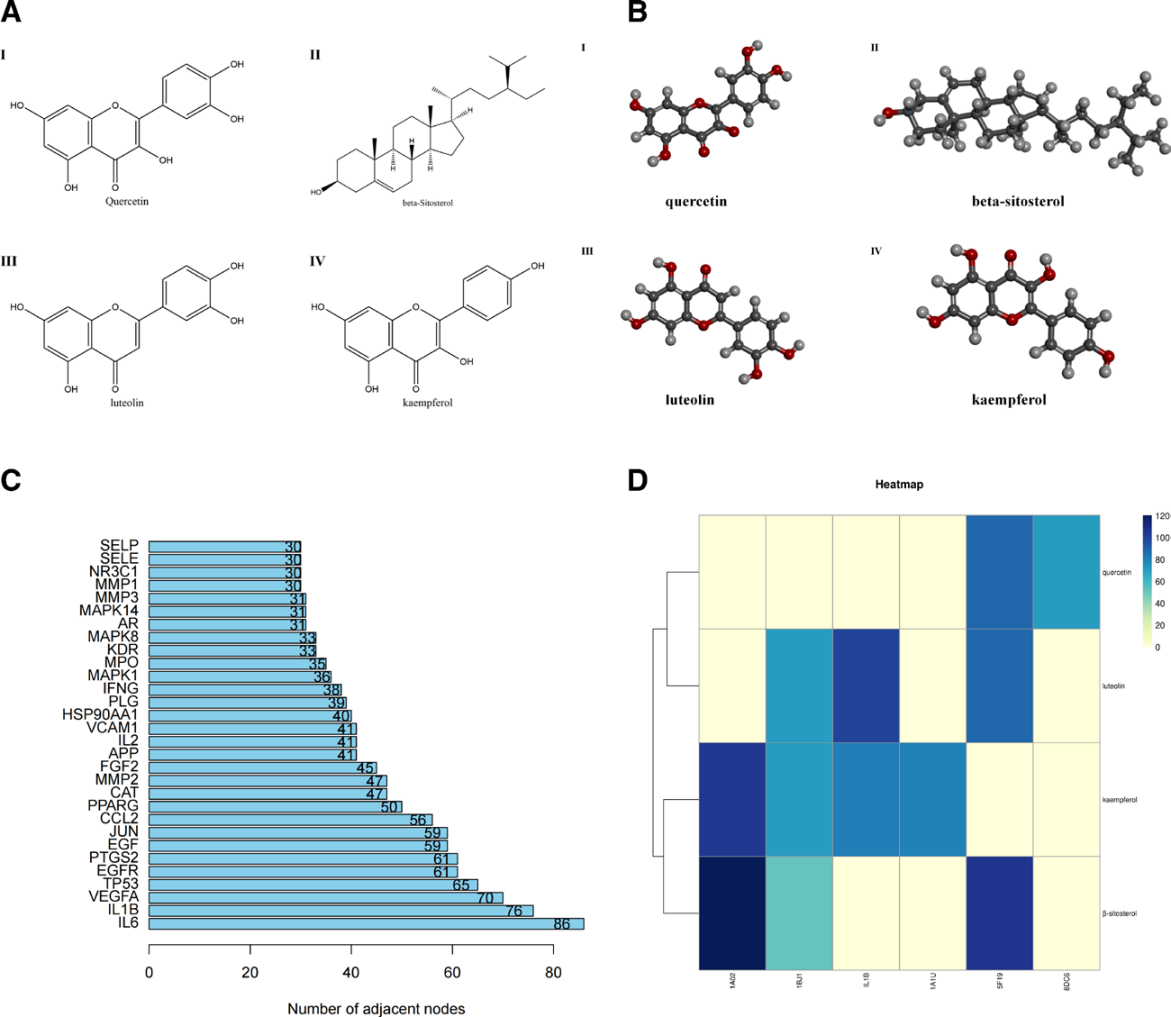
(A) Bar diagram of corresponding core targets genes of 4 main ingredients based on the degree value. (B) Heatmap of main active ingredients of *PV* with 6 core target genes.

**Figure 5.**
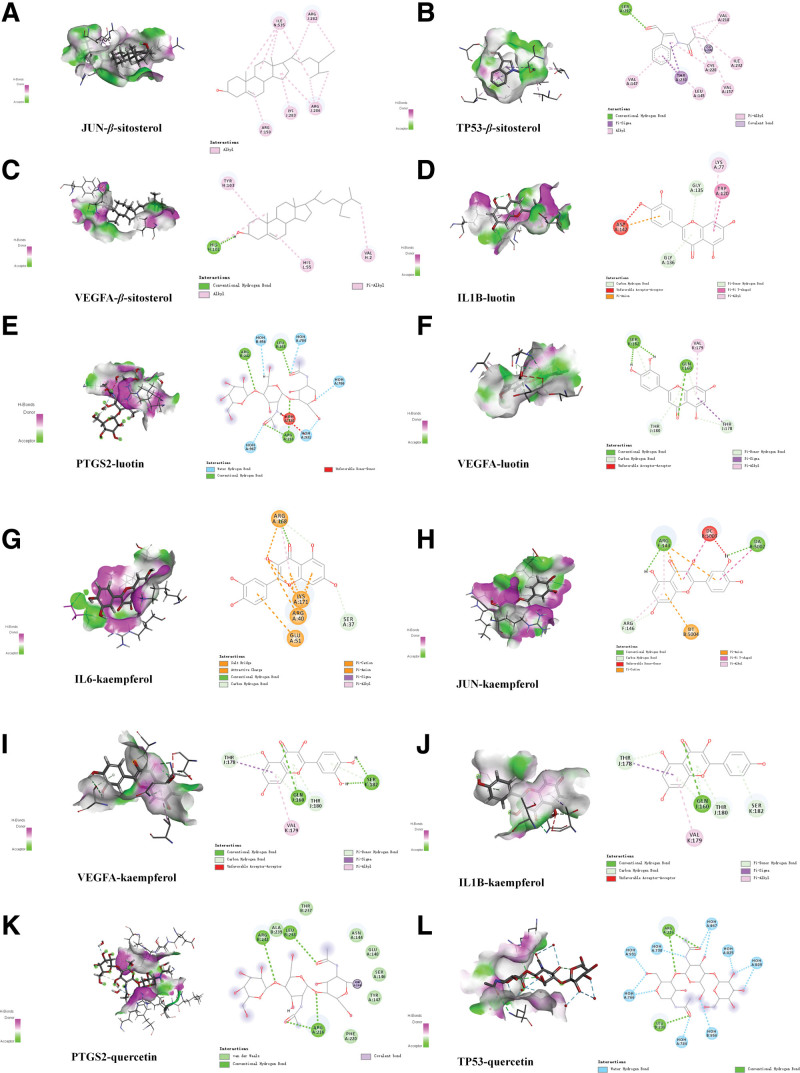
Detailed “targets-drug” interactions of the molecular docking verification. (A) β-sitosterol JUN, (B) β-sitosterol-TP53, (C) β-sitosterol VEGFA, (D) luteolin-IL1B, (E) luteolin-PTGS2, (F) luteolin VEGFA, (G) kaempferol-IL6, (H) kaempferol JUN, (I) kaempferol VEGFA, (J) kaempferol-IL1B, (K) quercetin-PTGS2, and (L) quercetin-TP53. β-sitosterol = beta-sitosterol, IL1B = recombinant human interleukin 1 beta, IL6 = interleukin 6, JUN = transcription factor AP-1, PTGS2 = prostaglandin endoperoxidase 2, TP53 = tumor protein p53, VEGFA = vascular endothelial growth factor A.

The 2-dimensional and 3-dimensional structural diagrams of the 4 active compounds of *PV* were presented in Figure 4A and Figure 4B respectively. Based on the PPI network, we selected the top 6 of degree as the core targets for molecular docking with 4 active compounds of *PV* (Fig. 4C). It is mainly combined with the target through traditional hydrogen bond, hydrocarbon bond, Pi-Pi conjugation, etc. In addition, the libdock score of each targets and compounds are presented in Figure 4D. Libdock score indicates the binding degree of ligand and target protein crystal. The higher the value is, the stronger the molecular binding ability is. Among them, those with the strongest combination ability with JUN are β-sitosterol (120.529) (Fig. 5A) and kaempferol (104.874) (Fig. 5H), the strongest binding ability with VEGFA are luteolin (71.465) (Fig. 5F), β-sitosterol (52.1039) (Fig. 5C) and kaempferol (71.3777) (Fig. 5I), the strongest binding ability with recombinant human interleukin 1 beta (IL1B) are luteolin (100.753) (Fig. 5D) and kaempferol (80.8784) (Fig. 5J), the strongest binding ability with IL6 is kaempferol (79.3898) (Fig. 5G), the binding ability with PTGS2 are luteolin (88.4373) (Fig. 5E) and quercetin (106.552) (Fig. 5K), the binding ability with TP53 is quercetin (71.63) (Fig. 5L) and β-sitosterol (106.552) (Fig. 5B) respectively. It was revealed that the 4 core components of *PV* exhibit good binding with PTC related targets respectively (Table 3, Fig. 5).

**Table 3:** The results of molecular docking.

Compound	Target	PDB	Libdock score
Quercetin	PTGS2	5F19	88.3876
Quercetin	TP53	8DC6	71.63
Luteolin	VEGFA	1BJ1	71.465
Luteolin	IL1B	1H1B	100.753
Luteolin	PTGS2	5F19	88.4373
Kaempferol	JUN	1A02	104.874
Kaempferol	VEGFA	1BJ1	71.3777
Kaempferol	IL1B	1H1B	80.8784
Kaempferol	IL6	1A1U	79.3898
Β-sitosterol	JUN	1A02	120.529
Β-sitosterol	VEGFA	5F19	52.1039
Β-sitosterol	TP53	8DC6	106.552

β-sitosterol = beta-sitosterol, IL1B = recombinant human interleukin 1 beta, IL6 = interleukin 6, JUN = transcription factor AP-1, PDB = protein data bank, PTGS2 = prostaglandin endoperoxidase 2, TP53 = tumor protein p53, VEGFA = vascular endothelial growth factor A.

### 3.7. Experimental verification results

#### 3.7.1. The IC_50_ values of active compounds in PV.

**Figure 6.**
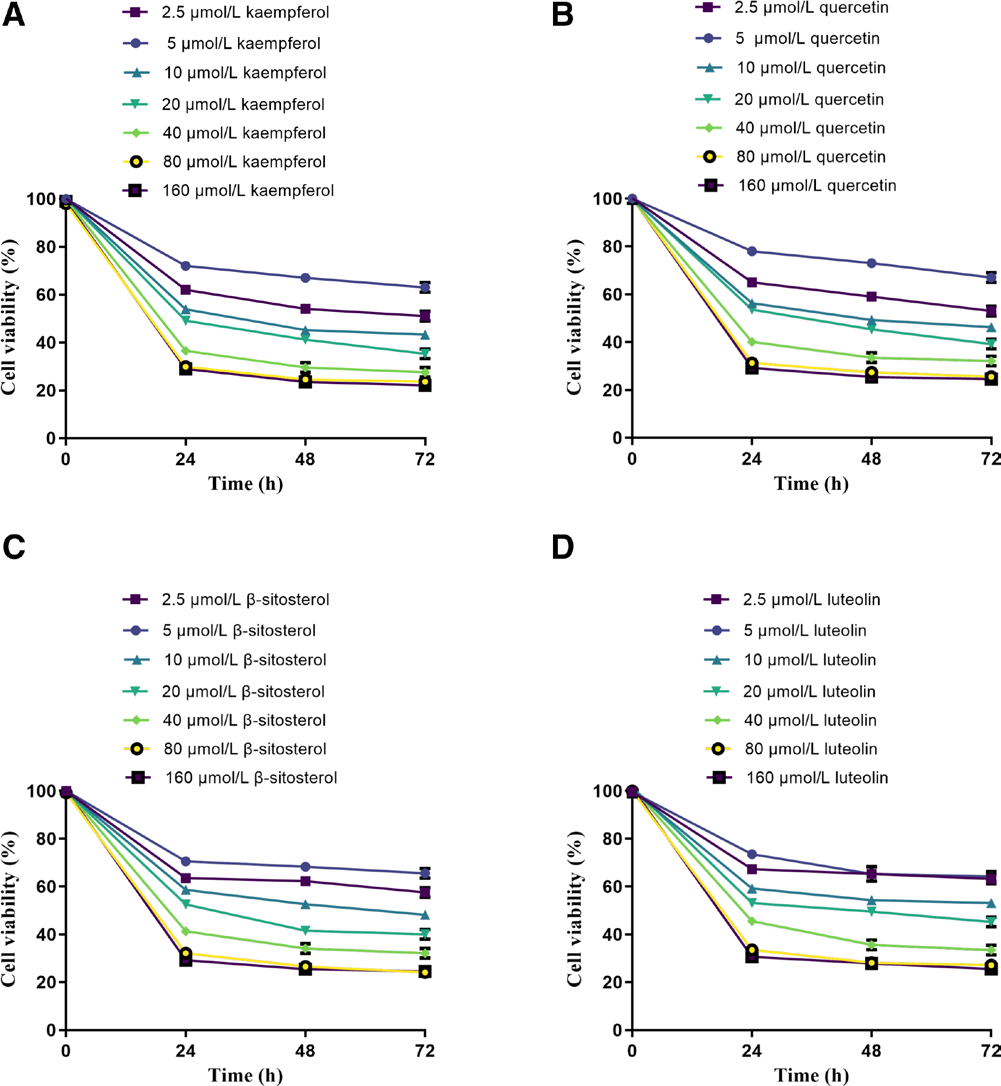
The cell viability of 4 main ingredients of *PV* on BCPAP cell. (A) kaempferol, (B) quercetin, (C) β-sitosterol, (D) luteolin. BCPAP = Papillary cells of human thyroid carcinoma bcpap cell lines, β-sitosterol = beta-sitosterol.

The active compounds quercetin, luteolin, β-sitosterol, and kaempferol from *PV* with different concentrations showed certain inhibition rates on BCPAP cells after 24, 48, and 72 hours of treatment, respectively. The IC_50_ values of quercetin at 24, 48, and 72 hours were 0.361, 0.302, 0.291 µmol/mL respectively (Fig. 6A). The IC_50_ values of luteolin at 24, 48, and 72 hours were 0.425, 0.383, and 0.312 µmol/mL respectively (Fig. 6B). The IC_50_ values of β-sitosterol at 24, 48, and 72 hours were 0.361, 0.323, and 0.297 µmol/mL respectively (Fig. 6C). The IC_50_ values of kaempferol at 24, 48, and 72 hours were 0.311, 0.285, 0.262 µmol/mL respectively (Fig. 6D). Among them, kaempferol showed the strongest inhibition on the growth of BCPAP cells (IC_50_ value was the lowest). Therefore, kaempferol was selected as the most effective compound of *PV* in subsequent experiments. It was revealed that kaempferol has a significant time effect relationship and a dose effect relationship on the inhibition of BCPAP cells, and the inhibition rate is the highest.

#### 3.7.2. Effect of kaempferol on the expression of PTC related proteins

**Figure 7.**
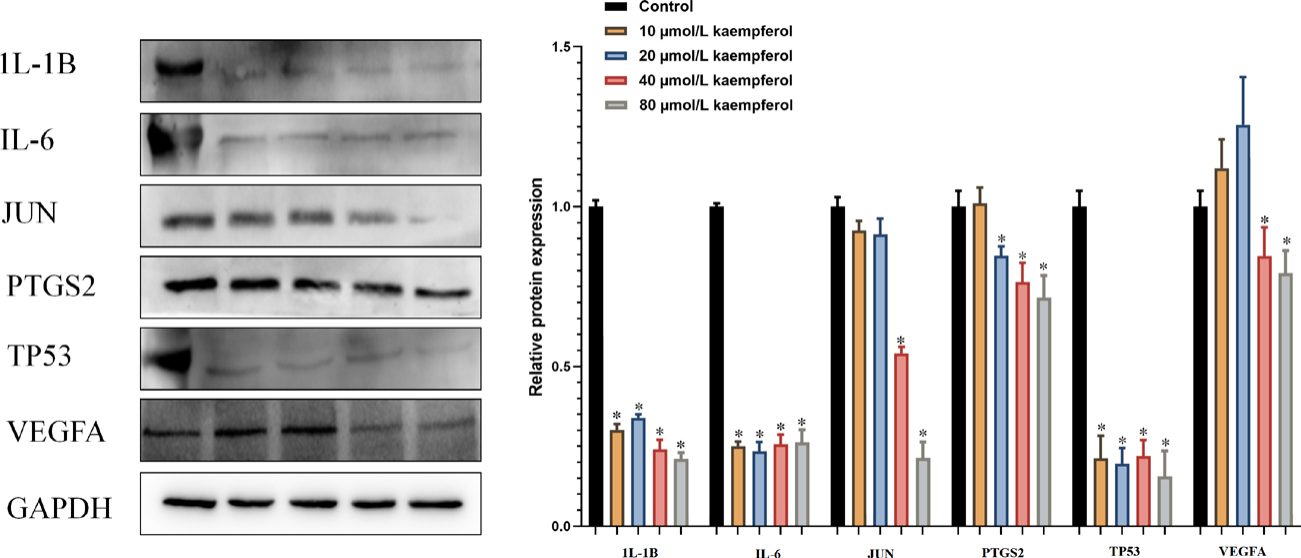
Effect of kaempferol on the expression of related proteins of PTC. (A) IL-1B, (B) IL6, (C) JUN, (D) PTGS2, (E) TP53, (F) VEGFA, IL6 = interleukin 6, JUN = transcription factor AP-1, PTC = papillary thyroid cancer, PTGS2 = prostaglandin endoperoxidase 2, TP53 = tumor protein p53, VEGFA = vascular endothelial growth factor A.

In order to explore the mechanism of *PV* on anti-PTC, the protein expression levels of VEGFA, TP53, JUN, PTGS2, IL6, and IL-1B were detected respectively in the present study (Fig. 7). Compared with the control group, kaempferol at concentrations of 10, 20, 40, 80 µmol/L significantly decreased the expression of 1L-1B, IL6, and TP53 protein respectively (*P*<.05). Kaempferol could significantly reduce the expression of PTGS2 protein at concentrations of 20, 40, 80 µmol/L. In addition, kaempferol may significantly reduce the expression of JUN and VEGFA protein at concentrations of 40, 80 µmol/L respectively (*P*<.05).

## 4. Discussion

PTC is the most common endocrine system tumor, which belongs to the category of “goiter and tumor” disease in TCM which accounts for about 80% to 90% of DTC.^[14,15]^ TCM has shown great potential in the prevention and treatment of tumors and other diseases. The separation and extraction of effective components from TCM is an important means of new drug development.^[16]^ It is reported that more than 80% of small molecule antitumor drugs are natural products and their derivatives.^[17]^ The diverse pharmacological activities of natural compounds provide an important basis for the study of the mechanism of biological functions. Previous studies have shown that *PV* may inhibit the proliferation of tumor cells, induce apoptosis, and regulate immunity in many kinds of tumors.^[12]^ However, due to the complexity of the components of TCM, its main effective components and related mechanisms are still unclear. Network pharmacology is to use systems biology, high-throughput screening and other technologies to systematically analyze the interaction network of “TCM components and disease targets,” and to reveal the intervention and impact of TCM on body diseases as a whole, which is consistent with the theory of TCM treatment.^[18]^

In the present study, the main active components, targets and functional pathways of *PV* in the treatment of PTC were preliminarily explored by network pharmacology. The results showed that there were 11 main active ingredients of *PV* in the treatment of PTC, including β-sitosterol, kaempferol, spinasterol, stigmasterol, delphinidin, luteolin, vulgaxanthin-I, poriferasterol monoglucoside_qt, stigmast-7-enol, morin, quercetin. According to the analysis of the network of the “active ingredient-target-disease” with Cyto scape 3.7.2, the top 4 active compounds of *PV* are quercetin, luteolin, β-sitosterol, kaempferol. Quercetin is a unique bioactive flavone and antioxidant, which plays a full role in reducing different human cancers that can directly promote apoptosis of tumor cells, so it can inhibit the progression of many human cancers.^[19]^ It has been shown that quercetin can inhibit thyroid cancer cells in *vitro*.^[20]^ In addition, it was also reported that quercetin is effective in the treatment of both medullary and papillary of human thyroid cancer.^[21]^ β-sitosterol is the most abundant and widely distributed in vegetable foods rich in oil. It was found that prominent in *vitro* antiproliferative and pro-apoptotic effects of β-sitosterol in MDA-MB231 cells.^[22]^ Previous study revealed that the silver nanoparticles of β-sitosterol can effectively induce the toxicity and early apoptosis of human colon cancer cells by enhancing the expression of p53 protein.^[23]^ Luteolin may show effective cytotoxicity to thyroid cancer cells in vitro and in *vivo* by blocking BANCR/TSHR signals which may become a potential important anticancer agent for thyroid cancer progression.^[24]^ Meanwhile, luteolin exhibits an antitumor effect in the HCC cell line SMMC-7721 by suppress cell viability, induce G0/G1-phase arrest, and increase cellular apoptosis.^[25]^ Kaempferol may inhibited cellular growth in a dose dependent manner in F9 and thyroid cancer cells.^[26]^ It was reported that kaempferol inhibits the invasion and migration of renal cancer cells through the downregulation of AKT and FAK pathways.^[27]^ Taken together, the 4 core components of *PV* showed different levels of anticancer activity.

These active ingredients act on 83 target proteins of PTC. PPI network shows that the genes with the top 6 connectivity are VEGFA, TP53, JUN, PTGS2, IL6, and IL1B, respectively. It shows that these targets are related to the proliferation and differentiation of tumor cells and tumor progression in the present study. Among them, VEGFA is a key mediator of cancer-associated neo-angiogenesis and progression which have been explored extensively for cancer therapy. It was found that the importance of increased VEGFA protein in tumors and may explain the higher protein quantification observed in the PTC tumor samples in comparison to the goiter.^[28]^ It was also revealed that the molecular state of VEGFA may play an important role in the progression of PTC. VEGFA mRNA is overexpressed in thyroid carcinoma, especially in thyroid carcinoma with lymph node metastasis.^[29]^ Previous research results found that the role of TP53-rs1042522 polymorphism in development of thyroid carcinoma which related to decreased risk of PTC, smaller tumor size, and lower incidence of vascular invasion.^[30]^ In addition, it was indicated that TP53 may lead to the risk of PTC in individuals exposed to radiation in late childhood, adolescence or adulthood in previous study.^[31]^ It has been demonstrated that the gene JUN was closely connected with PTC genesis which may benefit the cure of PTC patients.^[32]^ It was found that the PTGS2 expression was significantly higher in patients with lymph node metastasis or extrathyroidal extension.^[33]^ IL6 plays an important role in the progression of thyroid cancer that targeting IL6 signal transduction may help the clinical treatment of patients with thyroid cancer with more invasive tumor characteristics.^[34]^ The IL6/JAK2/STAT3 pathway was considered to be associated with PTC malignant behaviors.^[35]^ IL1B polymorphism may be associated with PTC risk and a predictor of lymph node metastasis of PTC patients in Korean population.^[36]^ The results of molecular docking showed that the binding strength of key components corresponding to key targets was good. It further verified that *PV* may play a role in the treatment of PTC through these core pharmacodynamic components acting on the corresponding targets.

KEGG and GO analysis showed that the potential mechanism of *PV* in treating PTC mainly focused on cell proliferation, differentiation and anti-inflammatory immunity. The occurrence, development, malignant transformation, invasion, and metastasis of tumor are accompanied by inflammatory reaction. Inflammatory cells and molecules play a key role in the formation and maintenance of tumor immunity.^[37]^ It had been demonstrated that the level of IL6 in patients with thyroid cancer is elevated, and there is a close relationship between thyroid cancer and inflammation.^[38]^ The level of serum interleukin fully reflects this relationship and the potential mechanism of action. IL-17 is an proinflammatory cytokine cause continuous inflammatory reaction that increase the expression of nuclear factor kappa B, thus activating the expression of effector genes IL6 and vascular endothelial growth factor which promoting the occurrence and development of tumors.^[39]^ PI3K-AKT signal pathway is involved in cell proliferation, apoptosis, cycle regulation and other pathophysiological processes.^[40]^ P53 is one of the main apoptotic signal pathways, which can promote apoptosis by interacting with BCL-2 family proteins in the cytoplasm.^[41]^ *PV* may treat PTC through multiple pathways and multiple targets. Besides above genes and pathways, it was also reported that BRAF gene mutation^[42]^ and TERT promoter mutation^[15]^ were closely associated with PTC recurrence and prognosis which should focus on the key points research in the future.

## 5. Conclusion

Eleven components and 83 corresponding targets were obtained in the component target network of *PV*, of which 6 were the core targets of *PV* in the treatment of PTC. Quercetin, luteolin, β-sitosterol, kaempferol may be the core components of *PV* in the treatment of PTC. VEGFA, TP53, JUN, PTGS2, IL6, and IL-1B were considered to be the targets for the treatment of PTC. IL-17 signaling pathway and PI3K-Akt signaling pathway may affect the recurrence and metastasis of PTC. In conclusion, this study used network pharmacology and molecular docking technology to clarify that *PV* may play a role in the treatment of PTC through multiple components, multiple targets and multiple pathways. In addition, further *vivo* experiment was carried to verify it. Kaempferol is consider to be the most useful compound which may reduce the protein expression levels of IL6, VEGFA, JUN, TP53, 1L-1B, and PTGS2, respectively. This study laid a foundation for the in-depth study of the material basis and mechanism of action of *PV*, and provided a scientific basis for the clinical application of *PV*.

## Acknowledgment

We want to thank Yanling Zhou of Chongqing Three Gorges Medical College for her contribution in revision of this manuscript.

## Author contributions

**Conceptualization:** Yuanshe Huang, Jingxin Mao.

**Data curation:** Xiling Zhu, Jingxin Mao.

**Formal analysis:** Xiling Zhu, Xiaodong Wang.

**Funding acquisition:** Jingxin Mao and Yanlin Zou.

**Methodology:** Xiling Zhu, Yan Li, Xiaodong Wang, Yuanshe Huang, Jingxin Mao.

**Project administration:** Jingxin Mao.

**Software:** Xiling Zhu, Xiaodong Wang, Jingxin Mao.

**Supervision:** Yan Li.

**Validation:** Jingxin Mao

**Visualization:** Jingxin Mao.

**Writing – original draft:** Xiling Zhu, Jingxin Mao.

**Writing – review & editing:** Yuanshe Huang, Jingxin Mao, Yanlin Zou.
